# Expression of Myoglobin in Normal and Cancer Brain Tissues: Correlation With Hypoxia Markers

**DOI:** 10.3389/fonc.2021.590771

**Published:** 2021-04-30

**Authors:** Marwa E. Elsherbiny, Mohammed Shaaban, Rana El-Tohamy, Islam E. Elkholi, Olfat Ali Hammam, Mona Magdy, Joan Allalunis-Turner, Marwan Emara

**Affiliations:** ^1^ Department of Pharmacology and Toxicology, Ahram Canadian University, 6th of October, Egypt; ^2^ Center for Aging and Associated Diseases, Zewail City of Science, Technology and Innovation, 6th of October, Egypt; ^3^ Department of Pathology, Theodor Bilharz Research Institute, Giza, Egypt; ^4^ Department of Oncology, Faculty of Medicine & Dentistry, University of Alberta, Edmonton, AB, Canada

**Keywords:** glioblastoma multiforme, myoglobin, human tissue microarray, lactate dehydrogenase A, carbonic anhydrase IX

## Abstract

**Background:**

Myoglobin (MB) is increasingly recognized as a key player in cancer growth and metastasis. Low oxygen tensions, commonly associated with highly aggressive and recurrent cancers, have been shown to regulate its expression in several cancers such as lung, neck, prostate and breast cancer. However, it is not yet known whether it contributes to the growth and spread of brain cancers especially Glioblastoma multiforme (GBM).

**Methods:**

Here we investigate the expression of MB, and its correlation with the hypoxia markers carbonic anhydrase IX (CAIX) and lactate dehydrogenase A (LDHA), in human tissue microarrays of multiple organ tumors, brain tumors, and GBM tumors, and their respective cancer-adjacent normal tissues. Correlation between MB protein expression and tumor grade was also assessed.

**Results:**

We show that MB protein is expressed in a wide variety of cancers, benign tumors, cancer-adjacent normal tissues, hyperplastic tissue samples and normal brain tissue, and low oxygen tensions modulate MB protein expression in different brain cancers, including GBM. Enhanced nuclear LDHA immune-reactivity in GBM was also observed. Finally, we report for the first time a positive correlation between MB expression and brain tumor grade.

**Conclusion:**

Our data suggest that hypoxia regulate MB expression in different brain cancers (including GBM) and that its expression is associated with a more aggressive phenotype as indicated by the positive correlation with the brain tumor grade. Additionally, a role for nuclear LDHA in promoting aggressive tumor phenotype is also suggested based on enhanced nuclear expression which was observed only in GBM.

## Introduction

Glioblastoma multiforme (GBM) is the most common primary brain cancer and is associated with poor prognosis (1). The recently reported 2- and 5-year survival rates of GBM patients are 26 - 33% and 4 - 5%, respectively (2). Pathognomonic features of GBM include the presence of microvascular proliferation and necrosis (3), both indicative of hypoxia. Since hypoxic cancer cells have a more aggressive phenotype, understanding the mechanisms responsible for cancer cell survival under hypoxic conditions could help identify factors responsible for GBM aggressiveness and recurrence (4, 5). Earlier reports show that GBM biopsies and xenograft derived cell lines display regional variation in oxygen consumption (6–8). Using cell lines from hypoxia-sensitive and hypoxia-tolerant GBM cells significant differences in their response to hypoxia were observed. The hypoxia-tolerant cell lines (M006x and M059K) were capable of reducing their oxygen consumption rates and maintaining their clonogenic potential after 4 days of hypoxia (0.6% oxygen) while the hypoxia-sensitive cells (M010b) failed to reduce their oxygen consumption rates and to maintain their clonogenic potential (6). These observations potentially explain GBM recurrence and ability to adapt to hypoxic tumor microenvironment, yet, the underlying hypoxia-adaptation mechanisms are still not clear.

The monomeric iron- and oxygen-binding protein, myoglobin (MB), was the first protein with a three-dimensional molecular structure to be reported (9, 10). MB has long been known to help in oxygen diffusion and storage and in both nitrite (NO2-) nitric oxide (NO) metabolisms in skeletal muscle and heart (11–13). Newer roles of MB are continuously being identified. For example, interaction with and possible aid in the intracellular transport of fatty acids as well as a potential role in cancer growth are being investigated (14).

MB expression has been reported in a wide variety of cancers such as human medullomyoblastoma and other non-myogenic epithelial cancers (15, 16). For example, its mRNA and protein were detected in a subset of breast cancer cell lines and is up-regulated in breast tumors compared to matched normal tissues (17, 18). Similarly, normal epithelial cells of the colon, ovary and lung were negative for MB while a subset of their respective tumors showed variable levels of MB expression (17). MB is also expressed in renal cell carcinoma and is up-regulated in prostate cancers compared to normal adjacent tissues (19, 20). Collectively these observations support a potential non-canonical role for MB in cancer growth.

In cancer, MB transcripts and/or protein are shown to be induced by different stimuli involved in cancer progression. For example, MB protein was induced by hypoxia, oxidative stress and exposure to epidermal growth factor in human MCF-7 breast cancer cell line, where oxidative stress was found to be the ultimate signal that triggers MB induction (17). In addition, MB expression significantly correlated with markers of hypoxia in a subset of invasive breast cancers (HIF-2α and CAIX) and in prostate cancer (CAIX) (18, 20). In line with these findings, MB expression in clear cell renal carcinoma correlated negatively with capillary density and positively with tumor grading with MB transcript and protein levels being induced under hypoxia in different renal carcinoma cell lines (19). It is worth mentioning that hypoxia-mediated MB up-regulation isn’t limited to cancer cells, in fact normal liver, brain, and gill of hypoxia-tolerant carp also up-regulate non-muscle type MB following five days of hypoxia (21).

We have previously reported that neuroglobin (Ngb) and cytoglobin (Cygb) are expressed in human GBM cells, various solid tumors, and various brain cancers and their corresponding normal adjacent tissues with hemoglobins (HB) being also expressed in human GBM cells (4, 22–25). Recently, we have reported that MB variants 2, 9, 10, 11, 13 are differentially expressed in GBM cells (26). In continuation of our previous work, we sought to investigate MB expression and its correlation with hypoxia markers: CAIX and lactate dehydrogenase A (LDHA), in human tissue microarrays (TMA) of multiple organ tumors, different brain tumors, and GBM tissue samples and their relative cancer-adjacent normal tissues (CANT). Our data suggest that in different brain malignancies’ including GBM, MB is regulated by hypoxia. We also detected an enhanced nuclear expression of LDHA in GBM. Contribution of these findings to the overall progression of different brain cancers, including GBM, is worthy of further exploration.

## Materials and Methods

### Tumor Tissue Microarray Immunostaining

Three groups of TMA slides, three slides each, were purchased from US Biomax Inc., Rockville, MD. These groups represent multiple organ tumors (Cat No. BC00432), brain tumors (Cat No. GL804), and GBM tumors (Cat No. GL805a), each with its relative cancer-adjacent normal tissue array. TMA slides were incubated in dry oven at 62°C for 2 hours, deparaffinized, dewaxed, rehydrated, washed, and then antigen retrieval was carried out using Bio SB TintoRetrieval Pressure Cooker (Bio SB Inc, CA) at high pressure for 15 minutes. TMA slides were washed, endogenous peroxidase activity was blocked with Dako REAL peroxidase blocking buffer (Code S2023, Dako Denmark A/S, Denmark) for 5 minutes, and then washed twice. TMA slides were blocked for 30 minutes at room temperature with 5% normal goat serum. Each TMA slide was incubated for 1 hour at room temperature with diluted (Dako antibody diluent, Code S0809, Dako Denmark A/S) primary antibodies against: MB [1:100 Rabbit anti-MB monoclonal antibody, clone EP3081Y, Cat. No. ab77232, abcam, MA], CAIX [1:150 rabbit anti-CAIX (D10C10) monoclonal antibody, Cat No. 5648, Cell signaling Technology, MA], and LDHA [1:100 rabbit anti-LDHA (C4B5) monoclonal antibody, Cat. No. 3582, Cell signaling Technology]. TMA slides were then washed, incubated with goat anti-rabbit immunoglobulin conjugated to peroxidase (Dako EnVision^+^, code K 4002, Dako Demark, A/S), and then visualized with Dako liquid DAB and substrate-chromagen system (Code No. K 3467, Dako Denmark A/S). The reaction was stopped by placing TMA slides in ddH_2_O. Slides were counterstained with Mayer’s hematoxylin (Code S3309, Dako Denmark A/S), rinsed in ddH_2_O. Thereafter slides were placed in lithium carbonate (Cat. No. 62470-100G-F, Sigma-Aldrich, MO) for a minute, washed, dehydrated, and then coverslips were mounted with Dako ultramount aqueous permanent mounting medium (Code S1964, Dako Denmark A/S).

Evaluation of TMA slides was performed by two independent pathologists (O.M. and M.M.) blinded to tissue specification data for all TMA slides. Each tissue core was assigned an overall score based on the percent positivity of both cytoplasm (c) and nuclear (n) as follows: 0/- (n/c) = absence of positively stained cells; 1/+ (n/c) = a few positive cells (<10%); 2/++ (n/c) = weak positive staining and occasional positive foci (10≥ and <25%); 3/+++(n/c) = intermediate staining and occasional positive foci (25≥ and < 50%); 4/++++ (n/c) = strongly positive in most of the sections and several positive foci (50≥ and <75%); and 5/+++++ (n/c)= strongly positive throughout with many intensely positive foci (≥75%).

### Statistical Analysis

Data are expressed as means ± standard error. Statistical analyses were performed using SigmaPlot 13 software (Systat Software Inc., Chicago, IL, USA). Differences between different tissues pathologies were assessed using One-way Analysis of Variance (ANOVA) then pairwise multiple comparison were made using Holm-Sidak method for multiple organ tumors TMA. For the other two TMA, different brain tumors and glioblastoma multiforme, multiple comparisons were made versus a control (normal brain tissue) using Dunn’s test. Additionally, Spearman Rank correlation analysis was used to assess the relationship between MB expression and the tested markers of hypoxia, CAIX and LDHA. The level of significance was set at p <0.05.

## Results

We screened each group of TMA: multiple organ tumors (39 cases/43 cores), different brain tumors (40 cases/80 cores), and GBM tumors (40 cases/80 cores), each with its respective CANT for CAIX, LDHA, and MB protein expression. Subcellular distribution of these proteins and correlations of their expression levels in these samples were assessed.

**Figure 1:**
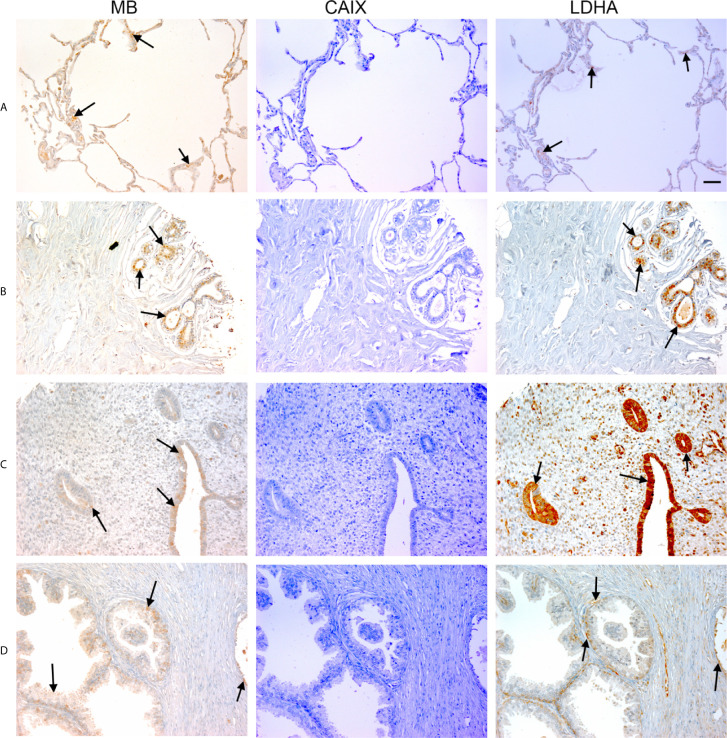
Expression of MB, CAIX, and LDHA in multiple organ tumor. Tissue microarrays containing cores obtained from multiple organ tumors and cancer adjacent normal tissues were stained with antibodies to MB, CAIX, and LDHA. All photomicrographs were obtained at 20× magnification and the scale bar indicates 50 μM. Black arrows indicate representative positive staining. **(A)** Cancer adjacent normal lung tissue with mild patchy cytoplasmic MB expression, negative CAIX, and focal weak cytoplasmic LDHA expression. **(B)** Cancer adjacent normal breast tissue with weak patchy cytoplasmic and focal nuclear MB expression, negative CAIX expression, and patchy moderate cytoplasmic, membranous and nuclear LDHA expression. **(C)** Cancer adjacent normal endometrial tissue with patchy weak to moderate cytoplasmic MB expression in the glands and occasional cytoplasmic in the endometrial stroma, negative CAIX expression in the glands and stroma, and diffuse moderate to strong cytoplasmic LDHA expression in the glands and mild to moderate cytoplasmic with patchy nuclear LDHA expression in the endometrial stroma. **(D)** Prostatic glands (hyperplasia) with focal mild cytoplasmic and membranous MB expression in the basal/myoepithelial cell layer only not in the luminal epithelial cells, negative CAIX expression in both basal and luminal cells, and patchy mild cytoplasmic and membranous LDHA expression in the basal/myoepithelial cell layer only not in the luminal epithelial cells.

**Figure 2:**
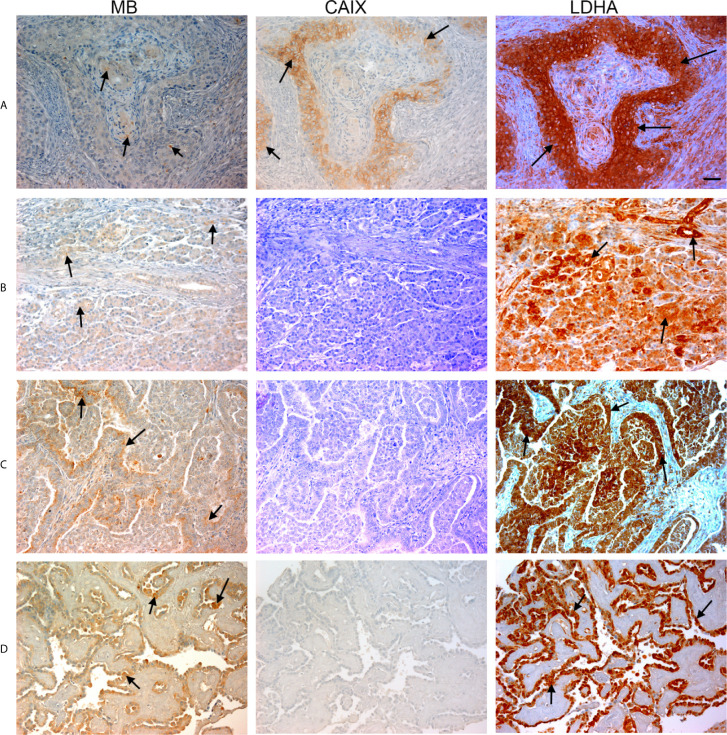
Expression of MB, CAIX, and LDHA in multiple organ tumor. Tissue microarrays containing cores obtained from multiple organ tumors and cancer adjacent normal tissues were stained with antibodies to MB, CAIX, and LDHA. All photomicrographs were obtained at 20× magnification and the scale bar indicates 50 μM. Black arrows indicate representative positive staining. **(A)** Squamous cell carcinoma (uterine cervix) with focal weak cytoplasmic MB expression, mild cytoplasmic and membranous CAIX expression, and strong diffuse cytoplasmic LDHA expression. **(B)** Duct adenocarcinoma (pancreas) with focal weak cytoplasmic MB expression in the acini, negative CAIX expression in the acini, and diffuse strong cytoplasmic and patchy membranous LDHA expression. **(C)** Adenocarcinoma of lung with patchy weak cytoplasmic MB expression in the acini, negative CAIX expression in the acini, and diffuse moderate to strong and patchy membranous and cytoplasmic LDHA expression. **(D)** Papillary carcinoma of thyroid with diffuse mild to moderate cytoplasmic MB expression in the acini, negative CAIX expression in the acini, and diffuse strong cytoplasmic LDHA expression.

Multiple organ tumor TMA contained samples from malignant tumors, benign tumors, hyperplasia and CANT (**Table 1**). Positive MB immunostaining was observed in all benign tumors (meningioma, ovarian serous papillary cystadenoma and adenomas of the thyroid and breast) and hyperplastic samples from cirrhotic liver and prostate. MB staining was also positive, and in a majority of malignant tumors (72%), mainly in thyroid papillary carcinoma, esophagus squamous cell carcinoma, nasopharyngeal carcinoma, hepatocellular carcinoma, breast invasive ductal carcinoma, kidney clear cell carcinoma, ovary serous cystadenocarcinoma, testis seminoma, skeletal muscle pleomorphic rhabdomyosarcoma, malignant melanoma of thigh, squamous cell carcinoma of scalp as well as adenocarcinomas of prostate, stomach, colon, pancreatic duct and lung. Likewise, MB immunostaining was positive in most (94%) CANT samples, specifically, in brain, lung, breast, kidney, ovary, bone marrow, skeletal muscle, and skin as well as CANT samples from different gastrointestinal organs (**Table 1**). Where expressed, MB localized mainly in the cytoplasm with few samples showing weak nuclear staining, mainly in normal tissues adjacent to lung, breast, and endometrial cancers, hyperplastic prostate tissue, and malignant melanoma of thigh (**Figures 1**, **2**). CAIX was positive only in a few malignant tumors (38%, mainly in nasopharyngeal carcinoma, lung adenocarcinoma and squamous cell carcinoma, kidney clear cell carcinoma, cervical squamous cell carcinoma, ovarian serous cystadenocarcinoma and malignant melanoma of thigh) and CANT (12.5%, mainly in stomach and skin) samples (**Table 1**). It was mainly localized to the cell membrane with a complete absence of nuclear staining (**Figures 1**, **2**).

**Table 1:** MB, CAIX, and LDHA expression in tissue microarrays of benign tumors, multiple organ tumors, hyperplasia, and cancer adjacent-normal tissue.

Organ	Pathology	Grade	MB(C/N)	LDHA(C/N)	CAIX(C/N)
**Brain** (Cerebrum)	Astrocytoma	II–III	0/-	0/-	0/-
	Meningioma	–	1/+	5/++	0/-
	CANT	–	5/+++	4/-	0/-
**Thyroid**	Papillary carcinoma	–	2/-	5/++++	0/-
	Adenoma	–	4/-	5/++	0/-
	CANT	–	0/-	0/+	0/-
**Esophagus**	Squamous cell carcinoma	II	3/-	5/+++	2/-
	CANT	–	1/-	1/+	0/-
**Nasopharynx** (Nose)	Nasopharyngeal carcinoma	–	3/+	2/-	3/-
**Stomach**	Adenocarcinoma	II	1/-	5/+++	0/-
	CANT	–	1/-	5/+++	5/-
**Colon**	Adenocarcinoma	II	3/-	5/++++	0/-
	CANT	–	4/++	5/+++	0/-
**Liver**	Hepatocellular carcinoma	II	2/-	5/++++	0/-
	Cirrhosis of liver	–	4/-	5/++	0/-
	CANT	–	5/-	5/+++++	0/-
**Pancreas**	Duct adenocarcinoma	–	3/-	5/+++	0/-
	CANT	–	3/-	1/-	0/-
**Lung**	Adenocarcinoma	I-II	3/-	5/++	1/-
	Squamous cell carcinoma	II	0/-	5/+++	2/-
	CANT	–	4/+	0/-	0/-
**Breast**	Invasive ductal carcinoma	II	5/-	5/++	0/-
	Adenoma	–	3/-	1/++	0/-
	CANT	–	5/++	3/+++	0/-
**Kidney**	Clear cell carcinoma	I	3/-	4/+++	3/-
	CANT	–	3/+	3/+	0/-
**Uterus**	Adenocarcinoma endometrium	II	0/-	5/++	0/-
**Uterine Cervix**	Squamous cell carcinoma	II	0/-	5/+++	3/-
	CANT	–	3/+	5/++	0/-
**Ovary**	Serous cystadenocarcinoma	II	3/-	5/+++	1/-
	Serous papillary cystadenoma	–	3/-	3/++	0/-
	CANT	–	1/-	0/-	0/-
**Prostate**	Adenocarcinoma	II	4/-	5/+++	0/-
	Hyperplasia	–	4/+++	1/-	0/-
**Testis**	Seminoma	–	1/-	2/-	0/-
**Bone**	Osteosarcoma (Femur)	–	0/-	3/+++	0/-
	CANT (Bone marrow)	–	5/	5/++	0/-
**Spleen**	NAT	–	1/	5/++	0/-
**Muscle**	Pleomorphic rhabdomyosarcoma	–	5/-	5/-	0/-
	CANT (Striated muscle)	–	4/-	4/-	0/-
**Skin**	Malignant melanoma of thigh	–	4/-	5/+++	3/-
	Squamous cell carcinoma of Scalp	II	3/-	5/++++	0/-
	CANT	–	3/-	5/++++	2/-
**Organ**	**Pathology**	**Grade**	**MB** **(C/N)**	**LDHA** **(C/N)**	**CAIX** **(C/N)**
**Brain** (Cerebrum)	Astrocytoma	II–III	0/-	0/-	0/-
	Meningioma	–	1/+	5/++	0/-
	CANT	–	5/+++	4/-	0/-
**Thyroid**	Papillary carcinoma	–	2/-	5/++++	0/-
	Adenoma	–	4/-	5/++	0/-
	CANT	–	0/-	0/+	0/-
**Esophagus**	Squamous cell carcinoma	II	3/-	5/+++	2/-
	CANT	–	1/-	1/+	0/-
**Nasopharynx** (Nose)	Nasopharyngeal carcinoma	–	3/+	2/-	3/-
**Stomach**	Adenocarcinoma	II	1/-	5/+++	0/-
	CANT	–	1/-	5/+++	5/-
**Colon**	Adenocarcinoma	II	3/-	5/++++	0/-
	CANT	–	4/++	5/+++	0/-
**Liver**	Hepatocellular carcinoma	II	2/-	5/++++	0/-
	Cirrhosis of liver	–	4/-	5/++	0/-
	CANT	–	5/-	5/+++++	0/-
**Pancreas**	Duct adenocarcinoma	–	3/-	5/+++	0/-
	CANT	–	3/-	1/-	0/-
**Lung**	Adenocarcinoma	I-II	3/-	5/++	1/-
	Squamous cell carcinoma	II	0/-	5/+++	2/-
	CANT	–	4/+	0/-	0/-
**Breast**	Invasive ductal carcinoma	II	5/-	5/++	0/-
	Adenoma	–	3/-	1/++	0/-
	CANT	–	5/++	3/+++	0/-
**Kidney**	Clear cell carcinoma	I	3/-	4/+++	3/-
	CANT	–	3/+	3/+	0/-
**Uterus**	Adenocarcinoma endometrium	II	0/-	5/++	0/-
**Uterine Cervix**	Squamous cell carcinoma	II	0/-	5/+++	3/-
	CANT	–	3/+	5/++	0/-
**Ovary**	Serous cystadenocarcinoma	II	3/-	5/+++	1/-
	Serous papillary cystadenoma	–	3/-	3/++	0/-
	CANT	–	1/-	0/-	0/-
**Prostate**	Adenocarcinoma	II	4/-	5/+++	0/-
	Hyperplasia	–	4/+++	1/-	0/-
**Testis**	Seminoma	–	1/-	2/-	0/-
**Bone**	Osteosarcoma (Femur)	–	0/-	3/+++	0/-
	CANT (Bone marrow)	–	5/	5/++	0/-
**Spleen**	NAT	–	1/	5/++	0/-
**Muscle**	Pleomorphic rhabdomyosarcoma	–	5/-	5/-	0/-
	CANT (Striated muscle)	–	4/-	4/-	0/-
**Skin**	Malignant melanoma of thigh	–	4/-	5/+++	3/-
	Squamous cell carcinoma of Scalp	II	3/-	5/++++	0/-
	CANT	–	3/-	5/++++	2/-

Three slides of tissue microarrays (TMA) of benign tumors, multiple organ tumors, hyperplasia, and cancer adjacent-normal tissue were purchased from US Biomax Inc., Rockville, MD. (Cat No. BC00432). TMA slides were stained with MB, CAIX, and LDHA antibodies and immunopositive staining was assessed by two pathologists. Each tissue core was assigned an overall score based on the percent positivity of both cytoplasm (C) and nuclear (N) as follows: 0/- (C/N) = absence of positively stained cells; 1/+ (C/N) = a few positive cells (<10%); 2/++ (C/N) = weak positive staining and occasional positive foci (10≥ and <25%); 3/+++ (C/N) = intermediate staining and occasional positive foci (25≥ and < 50%); 4/++++ (C/N) = strongly positive in most of the sections and several positive foci (50≥ and <75%); and 5/+++++ (C/N) = strongly positive throughout with many intensely positive foci (≥75%). CANT, Cancer adjacent-normal tissue.

Positive LDHA immunostaining was observed in all benign tumors and hyperplastic samples, and in the majority of malignant tumors (95%) and CANT (81%) samples (**Table 1**). A great deal of overlap between LDHA and MB positive samples was seen in malignant tumors apart from lung Squamous cell carcinoma, adenocarcinoma endometrium, cervical squamous cell carcinoma, osteosarcoma of the femur where LDHA immunostaining was positive while MB was negative. Similarly, LDHA immunostaining was positive in almost all MB positive tissue samples except lung and ovary CANT samples where LDHA immunostaining was negative while MB was positive. Where positive, LDHA showed a pattern of localization that was somewhat similar to that of MB, being mainly observed in the cytoplasm with weak-moderate nuclear staining detected in most of the tested samples (**Figures 1**, **2**).

**Figure 3:**
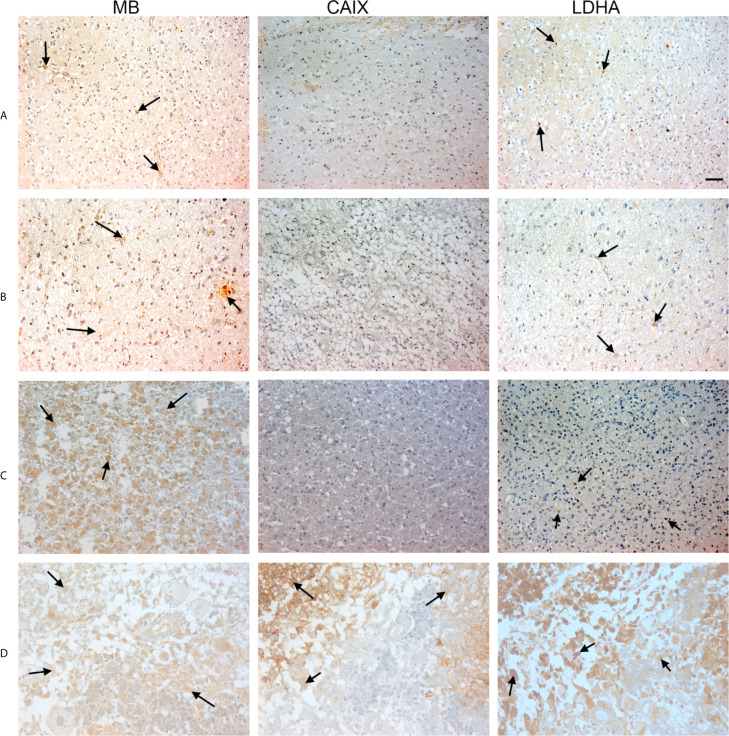
Expression of MB, CAIX, and LDHA in brain tumor. Tissue microarrays containing cores obtained from brain tumors, cancer adjacent normal brain tissues, and normal brain tissues were stained with antibodies to MB, CAIX, and LDHA. All photomicrographs were obtained at 20× magnification and the scale bar indicates 50 μM. Black arrows indicate representative positive staining. **(A)** Astrocytoma grade 1 with focal weak cytoplasmic MB expression, negative CAIX expression, and patchy weak cytoplasmic and occasional nuclear LDHA expression. **(B)** Astrocytoma grade 2 with diffuse mild cytoplasmic MB expression, negative CAIX expression, and occasional focal weak LDHA expression. **(C)** Astrocytoma grade 3 with diffuse moderate to strong cytoplasmic MB expression, negative expression of CAIX and occasional weak LDHA expression. **(D)** Glioblastoma with diffuse mild to moderate cytoplasmic MB expression, strong cytoplasmic and moderate membranous CAIX expression, and diffuse moderate to strong cytoplasmic LDHA expression.

Hypoxia markers (CAIX and LDHA) and MB protein expression was also investigated in a TMA of different brain tumors. The TMA contained tissue samples from malignant brain tumors (astrocytoma, protoplasmic astrocytoma, atypical astrocytoma, glioblastoma, oligodendroglioma, and anaplastic oligodendroglioma), CANT, and normal brain tissue. CAIX immunostaining was positive in the majority of malignant brain tumors (78%), but was reduced (10%) or absent from CANT and normal brain tissues, respectively (**Table 2**). Where expressed, CAIX was mainly detected in the cytoplasm with few samples showing membranous localization (3 glioblastomas and 1 oligodendroglioma) and weak nuclear staining (2 astrocytomas) (**Figure 3**). Significantly less cytoplasmic LDHA staining was observed in malignant tumors compared to normal brain tissue, which was equivalent to CANT (50% *vs.* 89% *vs.* 76%, respectively p = <0.001). This is in contrast to the significant increase in the percentage of cells with cytoplasmic CAIX staining observed in malignant brain tumors (25%) compared to normal brain tissue (0%) and CANT (3%) (p = <0.001). Although LDHA showed mainly cytoplasmic localization, weak-strong nuclear localization was also observed in malignant brain tumors, CANT, and normal brain tissues with no significant differences being detected in the percent of cells showing nuclear CAIX or LDHA staining (**Table 2** and **Figure 3**). MB protein expression was positive in the majority of malignant brain tumors (60%), and CANT (90%) and normal brain tissue samples (90%) (**Table 2**). MB immunostaining was mainly cytoplasmic with weak-strong nuclear localization being also observed in malignant tumors, CANT, and normal brain tissue (**Figure 3**).

**Table 2:** MB, CAIX, and LDHA expression in tissue microarrays of normal brain tissues, brain tumors, and cancer adjacent normal tissue.

Pathology/Brain	Grade	MB(C/N)	LDHA(C/N)	CAIX(C/N)
Astrocytoma	–	1/-	5/+	0/-
Astrocytoma	I-II	0/-	5/+	0/-
Astrocytoma	I	0/-	5/-	0/-
Astrocytoma	I	0/-	5/-	0/-
Astrocytoma	II	3/-	5/+++	0/-
Astrocytoma	II	3/-	5/+++	0/-
Astrocytoma	II	0/-	2/++	0/-
Astrocytoma	II	0/-	2/+	0/-
Astrocytoma	II	0/-	0/-	3/+++
Astrocytoma	II	0/-	0/-	5/+++
Astrocytoma	II	5/+++++	4/+++	5/+++
Astrocytoma	II	5/+++++	4/++++	5/++++
Astrocytoma	II	5/-	3/++	4/-
Astrocytoma	II	5/-	3/+	4/-
Astrocytoma	I	4/-	0/-	5/-
Astrocytoma	II	4/++	4/-	5/-
Astrocytoma	II	4/-	5/+++++	5/-
Astrocytoma	–	0/-	5/+++	5/-
Astrocytoma	II	0/-	4/-	1/-
Astrocytoma	II	0/-	4/-	1/-
Protoplasmic astrocytoma	II	0/-	2/++++	3/-
Protoplasmic astrocytoma	II	0/-	3/+++++	3/-
Astrocytoma	III	5/+++	5/+	3/-
Astrocytoma	III	5/+++	5/+	3/-
Astrocytoma	II	0/-	5/++++	1/-
Astrocytoma	II	0/-	5/+++	1/-
Astrocytoma	II	0/+	0/+	0/-
Astrocytoma	II	0/-	0/+	0/-
Astrocytoma	II	0/-	0/+	0/-
Astrocytoma	II	0/-	0/+	0/-
Astrocytoma	II-III	0/-	4/++	1/-
Astrocytoma	II-III	0/-	4/++	1/-
Astrocytoma	II	0/-	0/+	1/-
Astrocytoma	II	0/-	0/+	1/-
Atypical astrocytoma	III	5/+++	4/+++	3/-
Atypical astrocytoma	III	5/+++	4/+++	3/-
Glioblastoma	III-IV	5/+++	3/++++	2/-
Glioblastoma	III-IV	3/+++	5/+++	2/-
Glioblastoma	III-IV	3/+++	2/+	2/-
Glioblastoma	III-IV	3/+++	1	2/-
Glioblastoma	IV	5/++	5	3/-
Glioblastoma	IV	3/++	3	3/-
Glioblastoma	–	5/++	5	3/-
Glioblastoma	–	3/+++	5	3/-
Glioblastoma	IV	5/-	5	3/-
Glioblastoma	IV	5/-	4	5/-
Glioblastoma	IV	5/+	5	4/-
Glioblastoma	IV	5/+	4	3/-
Glioblastoma	IV	5/-	5	2/-
Glioblastoma	IV	5/-	5	1/-
Glioblastoma	IV	5/-	5	1/-
Glioblastoma	IV	5/-	5	2/-
Oligodendroglioma	I	5/-	0	1/-
Oligodendroglioma	–	5/-	0	0/-
Oligodendroglioma	II	4/-	0	0/-
Oligodendroglioma	II	4/+	4	0/-
Oligodendroglioma	II-III	4/-	5	2/-
Oligodendroglioma	II-III	4/-	5	1/-
Anaplastic oligodendroglioma	–	0/-	0	1/-
Anaplastic oligodendroglioma	–	4/-	1	1/-
CANT	–	5/++	5	0/-
CANT	–	5/++	5	0/-
CANT	–	5/-	4	0/-
CANT	–	5/-	4	0/-
CANT	–	0/-	5	0/-
CANT	–	4/-	5	3/-
CANT	–	5/+	4	0/-
CANT	–	5/++	4	0/-
CANT	–	5/-	5	0/-
CANT	–	5/-	5	0/-
NBT	–	0/+	5	0/-
NBT	–	1/+	5	0/-
NBT	–	5/-	5	0/-
NBT	–	5/-	5	0/-
NBT	–	5/+++	5	0/-
NBT	–	5/+++	5	0/-
NBT	–	4/++	5	0/-
NBT	–	4/++	5	0/-
NBT	–	4/++	5	0/-
NBT	–	4/++	5	0/-

Three slides of tissue microarrays (TMA) of normal brain tissues, brain tumors, and cancer adjacent normal tissue were purchased from US Biomax Inc., Rockville, MD. (Cat No. GL804). The TMA slides were stained with MB, CAIX, and LDHA antibodies and immunopositive staining was assessed by two pathologists. Each tissue core was assigned an overall score based on the percent positivity of both cytoplasm (C) and nuclear (N) as follows: 0/- (C/N) = absence of positively stained cells; 1/+ (n/c) = a few positive cells (<10%); 2/++ (n/c) = weak positive staining and occasional positive foci (10≥ and <25%); 3/+++ (C/N) = intermediate staining and occasional positive foci (25≥ and < 50%); 4/++++ (C/N) = strongly positive in most of the sections and several positive foci (50≥ and <75%); and 5/+++++ (C/N) = strongly positive throughout with many intensely positive foci (≥75%). CANT, Cancer adjacent normal tissue; NBT, Normal Brain Tissue.

**Figure 4:**
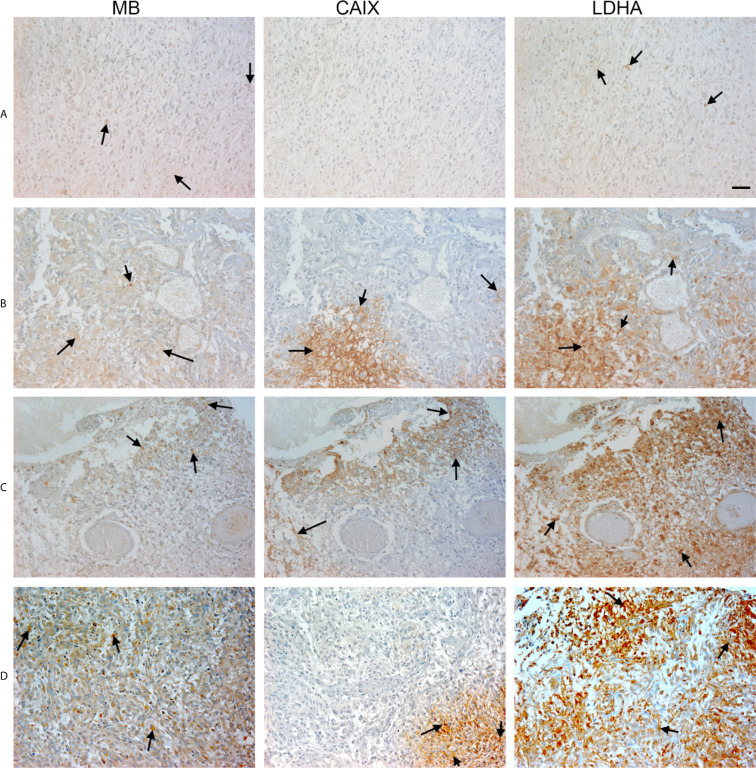
Expression of MB, CAIX, and LDHA in glioblastoma multiforme. Tissue microarrays containing cores obtained from glioblastoma tissues, cancer adjacent normal cerebrum tissues, and normal cerebrum tissue were stained with antibodies to MB, CAIX, and LDHA. All photomicrographs were obtained at 20× magnification and the scale bar indicates 50 μM. Black arrows indicate representative positive staining. **(A)** Glioblastoma Multiforme (cerebrum) with focal weak cytoplasmic MB expression, negative CAIX expression, and patchy weak cytoplasmic LDHA expression. **(B)** Glioblastoma Multiforme (cerebrum) with moderate cytoplasmic MB expression, patchy strong cytoplasmic CAIX expression, and moderate cytoplasmic and occasional nuclear LDHA expression. **(C)** Glioblastoma Multiforme (cerebrum) with patchy weak cytoplasmic MB expression, patchy moderate cytoplasmic CAIX expression, and moderate diffuse strong cytoplasmic and occasional nuclear LDHA expression. **(D)** Glioblastoma Multiforme with diffuse mild to moderate cytoplasmic MB expression, focal moderate cytoplasmic CAIX expression, and diffuse moderate to strong cytoplasmic and few nuclear LDHA expression.

**Figure 5:**
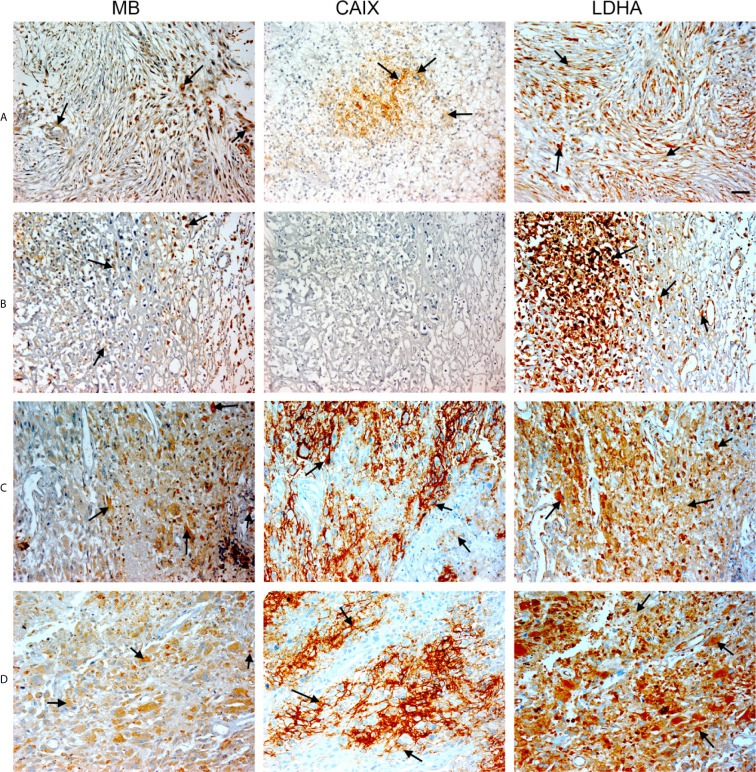
Expression of MB, CAIX, and LDHA in glioblastoma multiforme. Tissue microarrays containing cores obtained from glioblastoma tissues, cancer adjacent normal cerebrum tissues, and normal cerebrum tissue were stained with antibodies to MB, CAIX, and LDHA. All photomicrographs were obtained at 20× magnification and the scale bar indicates 50 μM. Black arrows indicate representative positive staining. **(A)** Glioblastoma Multiforme (cerebrum) with moderate cytoplasmic and few nuclear MB expression, focal mild to moderate cytoplasmic CAIX expression, and diffuse moderate cytoplasmic and nuclear LDHA expression. **(B)** Glioblastoma Multiforme (cerebrum) with patchy weak to moderate cytoplasmic MB expression, negative CAIX expression, and patchy moderate to strong cytoplasmic and nuclear LDHA expression. **(C)** Glioblastoma Multiforme (cerebrum) with patchy weak to moderate cytoplasmic MB expression, strong membranous and cytoplasmic CAIX, and moderate to strong cytoplasmic and few nuclear LDHA expression. **(D)** Glioblastoma Multiforme (cerebrum) with mild to moderate cytoplasmic MB expression, patchy moderate to strong membranous and cytoplasmic CAIX expression, and diffuse moderate to strong cytoplasmic and nuclear LDHA expression.

**Figure 6:**
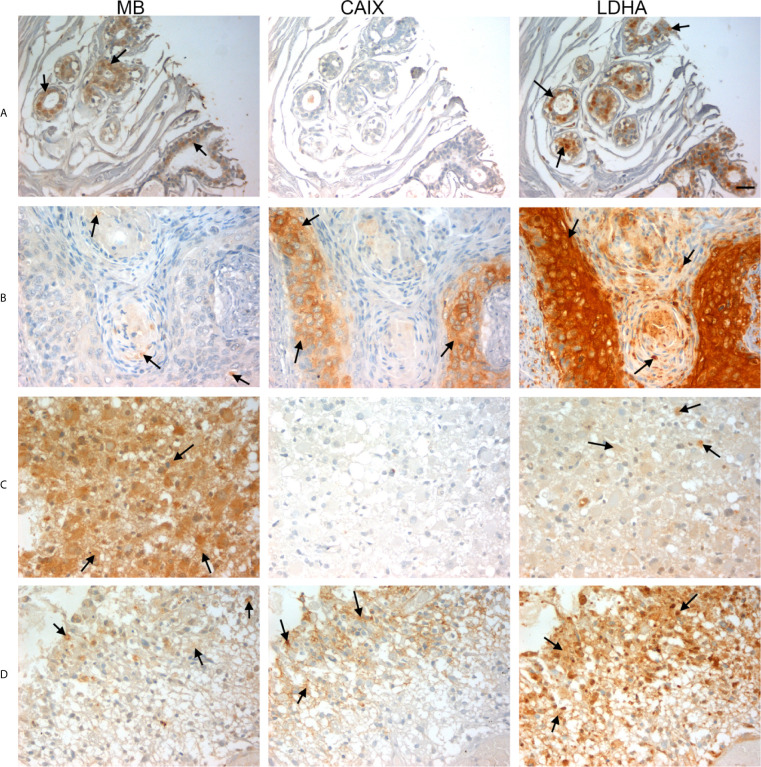
Expression of MB, CAIX, and LDHA in multiple organ tumor, brain tumor, and glioblastoma multiforme. All photomicrographs were obtained at 40× magnification and the scale bar indicates 25 μM. Black arrows indicate representative positive staining. **(A)** Cancer adjacent normal breast tissue with weak patchy cytoplasmic and focal nuclear MB expression, negative CAIX expression, and patchy moderate cytoplasmic, membranous and nuclear LDHA expression. **(B)** Squamous cell carcinoma (uterine cervix) with focal weak cytoplasmic MB expression, mild cytoplasmic and membranous CAIX expression, and strong diffuse cytoplasmic LDHA expression. **(C)** Astrocytoma grade 3 with diffuse moderate to strong cytoplasmic MB expression, negative expression of CAIX and occasional weak LDHA expression. **(D)** Glioblastoma Multiforme (cerebrum) with patchy weak cytoplasmic MB expression, patchy moderate cytoplasmic CAIX expression, and moderate diffuse strong cytoplasmic and occasional nuclear LDHA expression.

Next, we investigated CAIX, LDHA and MB protein expression in a TMA of GBM tumors, CANT, and normal brain tissue. CAIX immunostaining was positive in the majority of GBM samples but was absent from all CANT and normal brain tissue specimens (**Table 3**). Where expressed, CAIX mainly showed cytoplasmic and membranous localization. Additionally, a majority of GBM (98%), CANT (100%) and normal brain (67%) tissue samples were positive for LDHA (**Table 3**). Cytosolic localization of LDHA was observed in malignant, CANT and normal brain, however, the percentage of cells showing nuclear LDHA was significantly higher in GBM compared to normal brain (35% *vs.* 2%, p = <0.001, **Figures 4**, **5**, **Table 3**) with CANT samples showing a higher mean value than normal brain (27% *vs.* 2%, respectively, p = 0.062, **Figures 4**, **5**, **Table 3**). Finally, in the GBM TMA, there was no significant difference in the percentage cells positive for MB (cytoplasmic or nuclear) staining among the three tissue types. Immunostaining of MB, CAIX, and LDHA is shown in higher magnification in **Figure 6**).

**Table 3:** MB, CAIX, and LDHA in normal brain tissues microarrays of glioblastoma multiforme, and cancer adjacent normal tissue.

Pathology/Cerebrum	Grade	MB(C/N)	LDHA(C/N)	CAIX(C/N)
Glioblastoma	–	3/-	4/+++	1/+
Glioblastoma	III-IV	3/-	3/+++	1/+
Glioblastoma	IV	3/-	5/+++	2/+
Glioblastoma	IV	3/-	4/+++	2/+
Glioblastoma	IV	3/-	1/+	3/-
Glioblastoma	IV	3/-	1/+	3/-
Glioblastoma	IV	5/+++++	5/++	2/++
Glioblastoma	IV	5/+++++	4/++	3/++
Glioblastoma	III-IV	5/++++	5/++++	3/-
Glioblastoma	III- IV	4/++	5/+++	3/-
Glioblastoma	IV	3/-	1/+	3/-
Glioblastoma	IV	3/-	1/+	3/-
Glioblastoma	IV	3/-	4/+	0/-
Glioblastoma	IV	3/-	4/+	0/-
Glioblastoma	IV	5/-	4/++++	0/-
Glioblastoma	IV	5/-	4/+++	0/-
Glioblastoma	IV	5/++++	4/+++	3/-
Glioblastoma	IV	5/++++	3/+++	3/-
Glioblastoma	IV	0/-	5/++++	0/-
Glioblastoma	IV	0/-	5/+++	0/-
Glioblastoma	IV	3/-	1/+	3/-
Glioblastoma	IV	5/-	2/++	2/+
Glioblastoma	IV	3/-	1/++	2/-
Glioblastoma	IV	3/-	1/++	1/++
Glioblastoma	IV	5/+	5/++++	1/++
Glioblastoma	IV	5/+	5/+++++	2/++
Glioblastoma	IV	3/+	3/+++	0/-
Glioblastoma	IV	3/+	3/++	0/-
Glioblastoma	IV	2/-	5/+++++	2/++
Glioblastoma	IV	2/-	4/++++	0/-
Glioblastoma	IV	3/	4/+++	0/-
Glioblastoma	IV	5/+	4/++++	0/-
Glioblastoma	IV	4/+	5/++++	2/-
Glioblastoma	IV	3/+	5/+++	0/-
Glioblastoma	IV	5/-	3/+++	1/-
Glioblastoma	IV	5/-	3/+++	0/-
Glioblastoma	IV	5/+	3/+++	3/-
Glioblastoma	IV	5/++	3/+++	3/-
Glioblastoma	IV	5/++	3/+++	3/-
Glioblastoma	IV	5/++	3/+++	3/-
Glioblastoma	IV	5/-	5/++++	4/-
Glioblastoma	IV	5/-	5/+++	3/-
Glioblastoma	IV	3/-	5/+++	0/-
Glioblastoma	IV	5/-	5/+++	0/-
Glioblastoma	IV	5/-	3/+++	3/-
Glioblastoma	IV	5/-	4/+++	2/-
Glioblastoma	IV	5/-	4/++	3/-
Glioblastoma	IV	0/-	2/++	3/-
Glioblastoma (astrocytoma)	II	2/-	0/++	0/-
Glioblastoma (astrocytoma)	II	0/-	1/++	0/-
Glioblastoma	IV	3/-	2/++	1/-
Glioblastoma	IV	4/-	2/++	1/-
Glioblastoma (astrocytoma)	II	5/-	3/+	0/-
Glioblastoma (astrocytoma)	II	5/-	3/+	0/-
Glioblastoma	IV	5/+	5/+++++	4/-
Glioblastoma	IV	5/+	4/+++++	4/-
Glioblastoma	IV	5/-	4/++++	0/-
Glioblastoma	IV	5/-	5/++++	0/-
Glioblastoma	IV	5/++	5/++++	2/-
Glioblastoma	IV	5/++	5/++++	2/-
Glioblastoma	IV	2/-	5/+++++	1/-
Glioblastoma	IV	2/-	5/+++++	0/-
Glioblastoma	IV	4/+	4/+++	0/-
Glioblastoma	IV	4/-	4/++	1/-
Glioblastoma	IV	4/-	4/++	0/-
Glioblastoma	IV	4/-	4/++	1/-
Glioblastoma	IV	2/-	4/++	1/-
Glioblastoma	IV	2/-	5/+++++	1/-
Glioblastoma	IV	5/-	5/+++++	3/-
Glioblastoma	IV	5/-	5/++++	3/-
CANT	–	4/-	5/++	0/-
CANT	–	1/-	5/++	0/-
CANT	–	4/+++	5/+++	0/-
CANT	–	4/+++	5/+++	0/-
NCT	–	4/-	0/+	0/-
NCT	–	4/+	0/+	0/-
NCT	–	4/+	5/-	0/-
NCT	–	1/+	5/-	0/-
NCT	–	5/-	5/-	0/-
NCT	–	5/-	3/-	0/-

Three slides of tissue microarrays (TMA) of normal brain tissues, glioblastoma multiforme, and cancer adjacent normal tissue were purchased from US Biomax Inc., Rockville, MD. (Cat No. GL8045a). The TMA slides were stained with MB, CAIX, and LDHA antibodies and immunopositive staining was assessed by two pathologists. Each tissue core was assigned an overall score based on the percent positivity of both cytoplasm (C) and nuclear (N) as follows: 0/- (C/N) = absence of positively stained cells; 1/+ (n/c) = a few positive cells (<10%); 2/++ (n/c) = weak positive staining and occasional positive foci (10≥ and <25%); 3/+++ (C/N) = intermediate staining and occasional positive foci (25≥ and < 50%); 4/++++ (C/N) = strongly positive in most of the sections and several positive foci (50≥ and <75%); and 5/+++++ (C/N) = strongly positive throughout with many intensely positive foci (≥75%). CANT, Cancer adjacent normal tissue; NCT, Normal Cerebrum Tissue.

**Figure 7:**
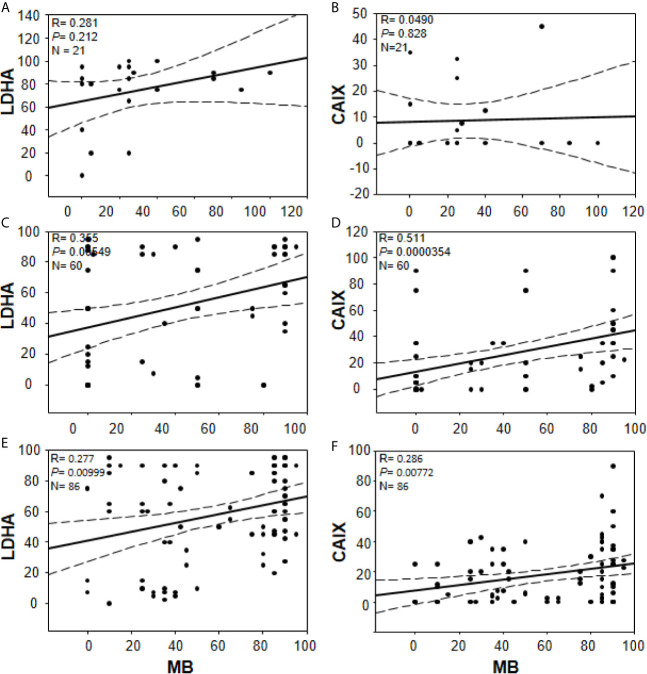
Correlations between percentage of positive cells for MB (x-axis, all panels) and LDHA (y-axis, panels **(A, C, E)** or CAIX y-axis, **(B, D, F)** in multiple organ tumors **(A, B)**, brain tumors **(C, D)**, and compiled glioblastoma of brain tumors and glioblastoma multiforme microarrays **(E, F)**. Dashed lines represent 95% confidence intervals.

**Figure 8:**
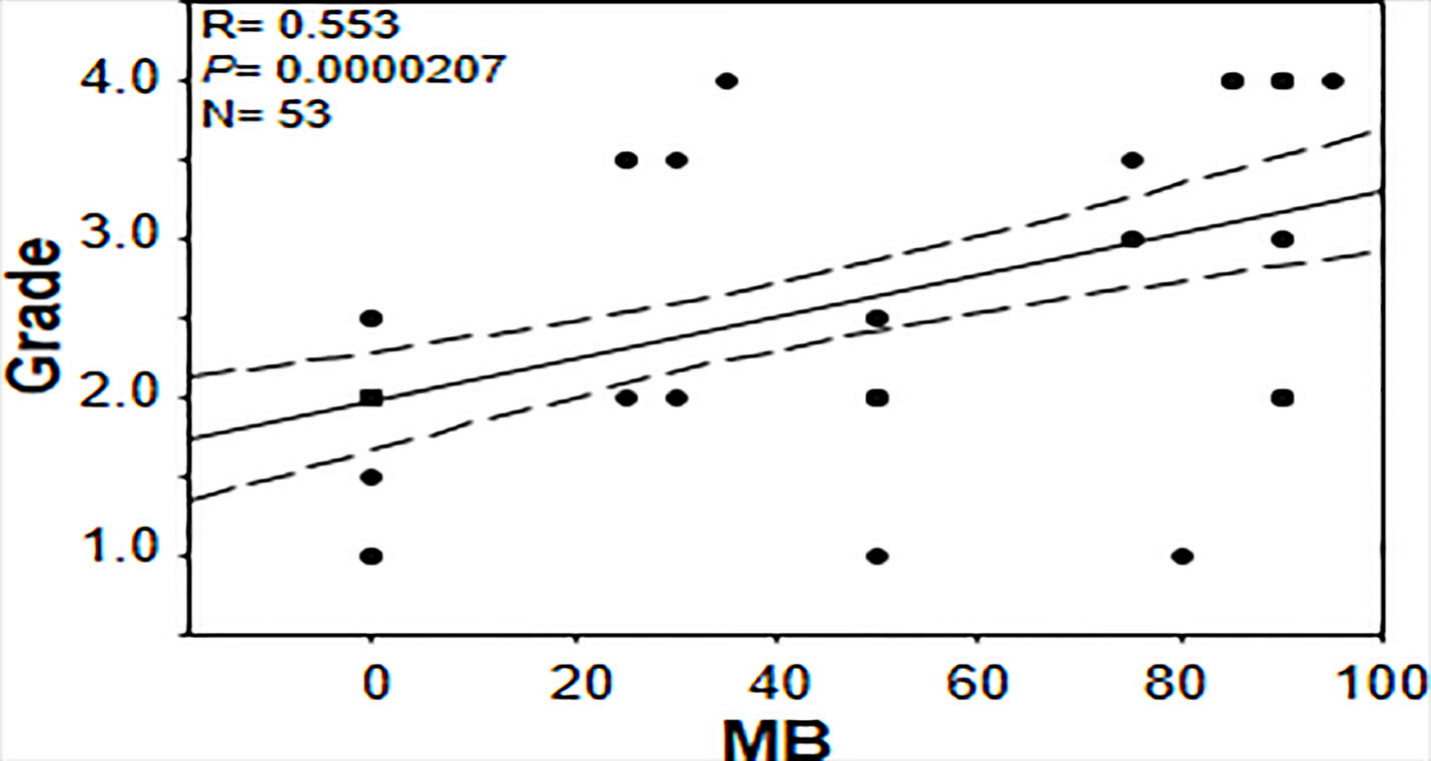
Correlation between percentage of positive cells for myoglobin and tumor grade in brain tumor tissue microarray.

In an effort to gain further insights into the regulation of tumor MB protein expression by hypoxia, Spearman Rank Order Correlation analysis of MB and both hypoxia markers (CAIX and LDHA) was carried out. Significant positive correlations between MB and LDHA (r = 0.355, p = 0.00549) and CAIX (r = 0.511, p = 0.0000354) immunostaining was observed in different malignancies of the brain (**Figure 7**). Similarly, MB immunostaining correlated positively with LDHA (r = 0.277, p = 0.00999) and CAIX (r = 0.286, p = 0.00772) in GBM samples (**Figure 7**). These results suggest that MB expression might be regulated by hypoxia in different brain malignancies including GBM. Intriguingly, MB immunostaining intensity showed a significant positive correlation with tumor grade in different brain malignancies (r = 0.553, p = 0.0000207, **Figure 8**). Collectively, these results suggest that MB is regulated, at least in part, by hypoxia and that its expression in different brain malignancies, including GBM, may be a marker of a more aggressive cancer phenotype as indicated by its positive correlation with tumor grade in different brain cancers.

## Discussion

Globins are expressed in different tumors and their expression has been shown to be altered under low oxygen tensions (4, 17, 19, 22, 24, 26). For example, it has been shown that Ngb and Cygb are expressed in GBM and are up-regulated by hypoxic conditions (4, 22). These observations potentially explain GBM’s remarkable ability to survive under hypoxia and the frequent recurrence rates associated with this cancer. Here, we confirm findings of other investigators that MB protein is expressed in a wide variety of cancers and we also report MB expression in some benign tumors, cancer-adjacent normal tissues, hyperplastic tissue samples and normal brain tissue. Our data also indicate that low oxygen tensions modulate MB protein expression in different brain cancers, including GBM. Enhanced nuclear LDHA immunoreactivity was readily detected in GBM; an observation that has not been previously reported. Finally, our results suggest that MB expression might serve as a marker of a more aggressive cancer phenotype, particularly in brain, as indicated by the positive correlation with the grade of different brain cancers.

Initially, we used a TMA containing a wide variety of cancers of different organs, benign tumors, CANT, and hyperplastic samples to generally assess MB expression and its correlation with markers of hypoxia. Using this highly variable array of samples, we could not detect a difference in MB protein expression or a correlation with known markers of hypoxia, CAIX and LDHA.

Consistent with earlier reports (27), we detected no CAIX expression in any of the normal brain tissues. Also, similar to an earlier report, we observed weak to absent CAIX immunostaining in astrocytoma and oligodendroglioma samples with GBM samples showing somewhat more intense (weak-strong) CAIX staining that localized mainly to cell membrane (27). In our study, CAIX expression was significantly associated with a malignant phenotype which is consistent with observations of other researchers who reported that CAIX expression increases with tumor grade (27–29). Together, these observations underline the contribution of this protein to the malignant phenotype.

LDHA localizes mainly in the cytoplasm exerting its lactate-producing function *via* the conversion of pyruvate to lactate and is up-regulated in many tumors (30, 31). Total LDHA expression has shown potential as a prognostic marker in some cancers such as clear cell renal cell carcinoma and gastric cancer (31, 32). Studies aimed at investigating subcellular localization of LDHA have reported cytoplasmic and nuclear localization of LDHA in some cancers such as esophageal squamous cell carcinoma. In fact in this cancer, high nuclear expression significantly correlated with advanced tumor stage and more lymph node metastases (33). The study also showed that the survival rate of patients with high expression of nuclear/cytoplasmic LDHA was significantly lower than that of patients with low expression with dual high expression of nuclear and cytoplasmic LDHA being associated with substantially lower survival rates than that of patients with single high or dual low nuclear and cytoplasmic LDHA. Interestingly, a recent article showed that reactive oxygen species (ROS) promote LDHA nuclear translocation in an immortalized human keratinocyte (HaCaT), human cervical cancer (HT-3), human bone osteosarcoma epithelial (U2OS), and HeLa cell lines where LDHA gains a noncanonical enzyme activity producing the antioxidant metabolite, α-hydroxybutyrate, potentially to protect cancer cells from ROS-mediated cell death (34). Consistent with these findings, in the different brain tumors TMA we observed a lower percent of cells with cytoplasmic LDHA staining compared to normal brain and a trend toward increased nuclear LDHA immunostaining in malignant brain tumors compared to normal brain. Further and in support of nuclear LDHA enhancement in malignant tumors, this pattern was also reproduced in our GBM TMA tissue microarray.

It should be noted that MB is expressed in normal human brain and other normal tissues (35–37) and different types of cancers and cell lines (17, 19–21, 26, 38–41). On the other hand, CAIX expression is restricted to a few normal tissues in the GIT (e.g. intrahepatic bile ducts, gastric mucosa, and duodenum) and is absent in the other normal tissues. It is differentially expressed in cancers with different expression patterns (e.g. diffuse, focal, membranous) (29, 42–46) and frequent expression is reported at the periphery of necrotic areas (42). Additionally, CAIX staining was not shown in all tissues of the same type of cancer or different type of cancers (43, 46). For example, the percentage of CAIX (IHC) positive cases in 1229 different types of cancer cases was about 30%, with 0.0%, 12.0%, 29.0%, and 0.0% CAIX staining being reported for prostate adenoma and carcinoma, pancreatic carcinoma, lung adenocarcinoma, and thyroid follicular adenoma, respectively (46). In contrast to CAIX, LDHA is expressed in normal tissues (47) and in many types of cancers, hypoxia-induced its expression (48–50). Based on this, it is quite expected not to see identical sites in the IHC images for MB and CAIX especially in normal tissues. However, we do see overlapping/similar pattern of staining for MB and CAIX in cancers and even more similar/identical sites were observed for MB and LDHA staining. Based on this, we assume that MB is partially regulated by hypoxia in brain cancers.

MB’s role in cancer is controversial with some reports advocate for a tumor suppressor role and others for a cancer survival strategy (19, 39, 51, 52). Association between MB expression and prolonged survival was observed in head and neck squamous cell carcinomas and breast cancer while correlation with poor survival and higher tumor grading was observed in lung cancer (in patients with adenocarcinoma) and clear cell renal carcinoma patients, respectively (18, 19, 39, 52). Using a forced expression approach in A549 human lung carcinoma cells, MB exerted a tumor suppressor role *via* reduction of the hypoxic response in spheroid cultures *in vitro* and in xeno-transplant mice models (51). The authors showed that Mb-mediated tumor oxygenation was the primary factor that promoted differentiation of cancer cells and suppressed both local and distal metastatic spreading because these effects were not observed using mutated forms of MB unable to bind oxygen and they were abrogated by the constitutive expression of an active form of HIF-1α (51). Using MB loss-of-function approach in stable knockdown MDA-MB468 cell line, increased oxygen uptake and elevated activities of mitochondrial enzymes during hypoxia, in addition to decreased cell viability and motility under both normoxic and hypoxic conditions were observed suggesting that MB also possesses unconventional functions that are not directly linked to its oxygen binding and transporting capacity (40). The authors assumed that MB potentially act as a shuttle for fatty acids thus supporting lipogenesis and cellular growth even at times when the oxygen supply is non-limiting (40). Consistent with previous reports supporting MB’s role in cancer cell survival, we not only detect significant positive correlations between MB and each of the tested markers of hypoxia in different brain cancers and in GBM, but we also detect a positive correlation between MB immunostaining and tumor grade (**Figures 5**, **7**). These results suggest that MB expression possibly mediates a more aggressive cancer phenotype, particularly in the brain.

Of note, this report is the first to show MB protein expression in normal human brain with neuroglobin, cytoglobin and hemoglobin already known to be expressed in normal brain (53). In fact, it has long been known that non-muscle MB is expressed in brains of hypoxia tolerant species such as hypoxia-tolerant carp, lungfish and subterranean mole rat Spalax (21, 54, 55) however its exact role is yet to be determined. Interestingly, it has been shown that the total Mb copy numbers in the *P. annectens* skeletal muscle and heart were lower than the brain, in the order of a tenth or less. Thus, in lungfish, it is considered that the brain is the main site of Mb expression not the heart or skeletal muscles and it was thought that MB might be vital in supplying oxygen to the nervous system during aestivation periods (54). Aside from human brain, we found reports showing MB protein expression in adult hematopoietic stem/progenitor cells obtained from peripheral or bone-marrow blood (56) and glandular cells of breast (18) and prostate (20). Studies aimed at exploring how human brain MB expression compares to human heart and skeletal muscles and its role in normal brain physiology should be useful additions to our current knowledge of MB function.

Similar to our results here, positive correlations between MB and different markers of hypoxia were observed in different cancers. For example, MB mRNA expression correlated with HIF1α (r = 0.483) or VEGFa (r = 0.468) in lung cancer tissue samples (52). In prostate cancer, MB immunostaining positively correlated with CAIX (r = 0.246) (20) and in invasive breast cancer MB immunostaining positively correlated with CAIX (r = 0.286) and HIF-2 α (r = 0.293) (18). Although these correlations are generally weak to moderate, they are in agreement with our reported correlation coefficients both in direction and in magnitude and indeed suggest a partial regulation of MB by hypoxia in a wide variety of cancers including different brain tumors and GBM. Nevertheless, they also indicate that additional factors drive MB expression in these cancers. Furthermore, it is conceivable that these factors are likely common among different cancers and are not dependent on the type of cancer in question.

A relatively recent study has demonstrated the existence of 19 splice variants for MB, of which variants 9, 10, 11, and 13 are protein-coding cancer-specific variants, and their expression was confirmed in MDA-MB468 breast cancer and DLD-1 colon cancer cell lines as well as in breast cancer biopsies (38). In these cell lines, the standard muscle-type MB transcript (variant 2) was also expressed, however in contrast to the cancer-specific variants (variants 9, 10 and 11) which were significantly induced, its expression was not affected by hypoxia (38, 40). Variants 9, 10, and 11 encode the standard MB protein as found in muscles (38). Based on this observation, it was assumed that the expression of these variants underlie the hypoxia-mediated increase in MB protein expression in breast cancer cell lines and tumor biopsies (38, 40). This assumption was also backed up by results from tumor biopsies where no correlation between MB variant 2 and VEGFA was detected, while significant positive correlations between VEGFA mRNA and MB variants 9, 10, 11 mRNA were observed (38). Likewise, we assume that the positive correlations observed herein between MB and markers of hypoxia at the protein level are potentially affected by MB expression from cancer-related variants in different brain tumors and GBM.

In summary we show that MB protein is expressed in a wide variety of cancers, benign tumors, cancer-adjacent normal tissues, hyperplastic tissue samples and normal brain tissue. Our data suggest that low oxygen tensions modulate MB protein expression in different brain cancers including GBM. We also detected enhanced nuclear immunoreactivity of LDHA in GBM that was not previously reported supporting a role for nuclear LDHA in promoting more aggressive tumor phenotype. Finally, MB expression is associated with a more aggressive cancer phenotype, specifically in brain cancers, as indicated by the positive correlation with the tumor grade.

## Data Availability Statement

The original contributions presented in the study are included in the article/**Supplementary Material**. Further inquiries can be directed to the corresponding author.

## Author Contributions

MEE: formal analysis, investigation, methodology, writing - original draft, and writing - review and editing. MS: methodology and writing - review and editing. RE-T: conceptualization, methodology, and writing - review and editing. IE: writing - review and editing. OH: data curation, methodology, visualization, and writing - review and editing. MM: data curation, methodology, visualization, and writing - review and editing. JA-T: conceptualization and writing - review and editing. ME: conceptualization, data curation, formal analysis, funding acquisition, investigation, methodology, project administration, resources, supervision, visualization, writing - review and editing. All authors contributed to the article and approved the submitted version.

## Funding

This research was funded by Zewail City for Science and Technology (internal fund), Giza, Egypt and Science and Technology Development Fund (STDF), grant number 12695, Ministry of Scientific Research, Egypt.

## Conflict of Interest

The authors declare that the research was conducted in the absence of any commercial or financial relationships that could be construed as a potential conflict of interest.

## Supplementary Material

Supplementary Document 1 Multiple organ tumor and adjacent normal tissue microarray.

Supplementary Document 2 Brain tumor and normal tissue microarray.

Supplementary Document 3 Glioblastoma multiforme and normal brain tissue microarray.
