# Supplementary material for: Expression of Myoglobin in Normal and Cancer Brain Tissues: Correlation With Hypoxia Markers
Source: Front Oncol. 2021 Apr 30;11:590771. doi: 10.3389/fonc.2021.590771 (PMC8120281; doi:10.3389/fonc.2021.590771)
Supplement: Supplementary Document 1 — Multiple organ tumor and adjacent normal tissue microarray. [file DataSheet_1.pdf]

# BC00432 : Multiple organ tumor and adjacent normal tissue array, 39 cases/43 cores

| | |
|---|---|
| Microarray Panel | Multiple organ tumor and adjacent normal tissue microarray, 18 types of tumor (bone marrow, breast, cerebrum colon, esophagus, kidney, liver, lung, nose, ovary, pancreas, prostate, skin, spleen, stomach, striated muscle, testis, thyroid and uterine cervix), single core per case |
| Cores | 43 |
| Cases | 39 |
| Row number | 6 |
| Column number | 8 |
| Core Diameter (mm) | 1 |
| Thickness (µm) | 5 |
| QA/QC | Anti-Actin confirmed |
| Tissue Array Type | FFPE |
| Species | Human |
| Applications | Routine histology procedures including Immunohistochemistry (IHC) and In Situ Hybridization (ISH), protocols which can be found at our support page. |
| Notes | 1. TMA slides were sectioned and stored at 4°C and may not be fresh cut, but still suitable for IHC. Please request fresh cut if experiment involves phospho-specific antibodies, RNA studies, FISH or ISH, etc. A minimum of 3 slides per TMA must be purchased to cover the cost of trimming for fresh sectioning. 2. Most TMA slides were not coated with an extra layer of paraffin (tissue cores can be easily seen on the glass). **To prevent tissue detachment during antigen retrieval, unbaked slides must be baked for at least 30 to 120 minutes at 60°C**. before putting into xylene for de-paraffinization. Baked slides were sent out baked for 2 hours. In the following specsheet,"*" means invalid core; "-" means no applicable or negative in IHC markers. |

BC00432 H&E

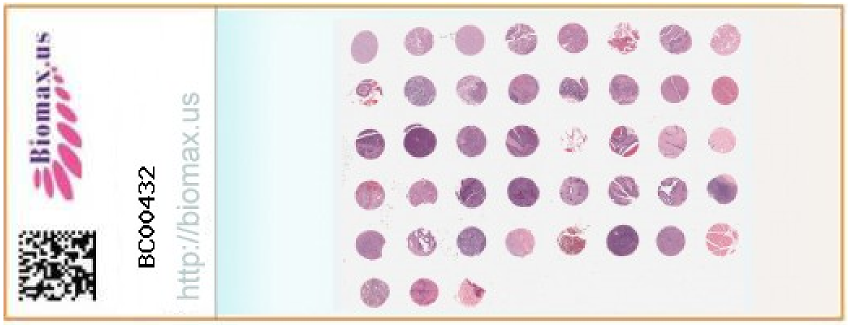


Mouseover and click individual cores to view high resolution images.

US Biomax, Inc. BC00432 (serial)

| | 1 | 2 | 3 | 4 | 5 | 6 | 7 | 8 |
|---|---|---|---|---|---|---|---|---|
| A | Cer | Cer | Cer | Thr | Thr | Thr | Eso | Eso |
| B | Nos | Sto | Sto | Col | Col | Liv | Liv | Liv |
| C | Pan | Pan | Lun | Lun | Lun | Bre | Bre | Bre |
| D | Kid | Kid | Ute | Ute | Ute | Ova | Ova | Ova |
| E | Pro | Pro | Tes | Bon | Bon | Spl | Str | Str |
| F | Ski | Ski | Ski | | | | | |

**Legend:Bon** - Bone marrow, **Bre** - Breast, **Cer** - Cerebrum, **Col** - Colon, **Eso** - Esophagus, **Kid** - Kidney, **Liv** - Liver, **Lun** - Lung, **Nos** - Nose, **Ova** - Ovary, **Pan** - Pancreas, **Pro** - Prostate, **Ski** - Skin, **Spl** - Spleen, **Sto** - Stomach, **Str** - Striated muscle, **Tes** - Testis, **Thr** - Thyroid, **Ute** - Uterine cervix

- Benign tumor, - Hyperplasia, - Malignant tumor, - NAT

, tissue IDs are available in exported Excel files.

| Pos. | No. | Age | Sex | Organ/Anatomic Site | Pathology diagnosis | TNM | Grade | Stage | Type | Image |
|---|---|---|---|---|---|---|---|---|---|---|
| A1 | 1 | 46 | F | Cerebrum | Astrocytoma | | 2--3 | | malignant | |
| A2 | 2 | 46 | M | Cerebrum | Meningioma | | - | | benign | |
| A3 | 3 | 39 | M | Cerebrum | Caner adjacent normal brain tissue | | - | | NAT | |
| A4 | 4 | 76 | F | Thyroid | Papillary carcinoam | | - | | malignant | |
| A5 | 5 | 28 | F | Thyroid | Adenoma | | - | | benign | |
| A6 | 6 | 48 | F | Thyroid | Cancer adjacent normal thyroid tissue | | - | | NAT | |
| A7 | 7 | 63 | F | Esophagus | Squamous cell carcinoma | | 2 | | malignant | |
| A8 | 8 | 63 | F | Esophagus | Cancer adjacent normal esophageal tissue (fi brous tissue and smooth muscle) of No. 07 | | - | | NAT | |
| B1 | 9 | 52 | M | Nose | Nasopharyngeal carcinoma | | - | | malignant | |
| B2 | 10 | 56 | M | Stomach | Adenocarcinoma | | 2 | | malignant | |
| B3 | 11 | 56 | M | Stomach | Cancer adjacent normal gastric tissue of No. 10 | | - | | NAT | |
| B4 | 12 | 66 | M | Colon | Adenocarcinoma | | 2 | | malignant | |

| | | | | | | | | | | |
|---|---|---|---|---|---|---|---|---|---|---|
| B5 | 13 | 66 | M | Colon | Cancer adjacent normal colonic tissue of N… 2 | | - | | NAT | |
| B6 | 14 | 56 | F | Liver | Hepatocellular carcinoma | | 3 | | malignant | |
| B7 | 15 | 38 | M | Liver | Cirrhosis of liver | | - | | hyperplasia | |
| B8 | 16 | 63 | M | Liver | Cancer adjacent normal hepatic tissue | | - | | NAT | |
| C1 | 17 | 49 | M | Pancreas | Duct adenocarcinoma (pancreatic tissue) | | - | | malignant | |
| C2 | 18 | 36 | F | Pancreas | Cancer adjacent normal pancreatic tissue | | - | | NAT | |
| C3 | 19 | 56 | F | Lung | Adenocarcinoma | | 1--2 | | malignant | |
| C4 | 20 | 58 | M | Lung | Squamous cell carcinoma | | 2 | | malignant | |
| C5 | 21 | 57 | M | Lung | Cancer adjacent normal pulmonary tissue | | - | | NAT | |
| C6 | 22 | 47 | F | Breast | Invasive ductal carcinoma | | 2 | | malignant | |
| C7 | 23 | 33 | F | Breast | Adenoma | | - | | benign | |
| C8 | 24 | 50 | F | Breast | Cancer adjacent normal breast tissue | | - | | NAT | |
| D1 | 25 | 38 | F | Kidney | Clear cell carcinoma | | 1 | | malignant | |
| D2 | 26 | 38 | F | Kidney | Cancer adjacent normal renal tissue of No. 25 | | - | | NAT | |
| D3 | 27 | 53 | F | Uterus | Adenocarcinoma endometrium | | 2 | | malignant | |
| D4 | 28 | 62 | F | Uterine cervix | Squamous cell carcnoma | | 2 | | malignant | |
| D5 | 29 | 42 | F | Uterine cervix | Cancer adjacent normal endometrial tissue | | - | | NAT | |
| D6 | 30 | 47 | F | Ovary | Serous cystadenocarcinoma | | 2 | | malignant | |
| D7 | 31 | 44 | F | Ovary | Serous papillary cystadenoma | | - | | benign | |
| D8 | 32 | 53 | F | Ovary | Cancer adjacent normal ovarial tissue | | - | | NAT | |
| E1 | 33 | 75 | M | Prostate | Adenocarcinoma | | 2 | | malignant | |
| E2 | 34 | 73 | M | Prostate | Hyperplasia | | - | | hyperplasia | |
| E3 | 35 | 32 | M | Testis | Seminoma | | - | | malignant | |
| E4 | 36 | 16 | M | Bone | Osteosarcoma of right femur | | - | | malignant | |
| E5 | 37 | 56 | M | Bone marrow | Cancer adjacent normal bone marrow tissue | | - | | NAT | |
| E6 | 38 | 9 | F | Spleen | Cancer adjacent normal splenic tissue | | - | | NAT | |
| E7 | 39 | 20 | M | Striated muscle | Pleomorphic rhabdomyosarcoma of left shoulder | | - | | malignant | |
| E8 | 40 | 50 | M | Striated muscle | Cancer adjacent normal skeletal muscle tissue | | - | | NAT | |
| F1 | 41 | 46 | F | Skin | Malignant melanoma of thigh | | - | | malignant | |
| F2 | 42 | 52 | M | Skin | Squamous cell carcinoma of scalp | | 1 | | malignant | |
| F3 | 43 | 49 | F | Skin | Cancer adjacent normal dermatic tissue | | - | | NAT | |

## TNM grading:

**T - Primary tumor**
Tx - Primary tumor cannot be assessed
T0 - No evidence of primary tumor
Tis - Carcinoma in situ; intraepithelial or invasion of lamina propria
T1 - Tumor invades submucosa
T2 - Tumor invades muscularis propria
T3 - Tumor invades through muscularis propria into subserosa or into non-peritonealized pericolic or perirectal tissues.
T4 - Tumor directly invades other organs or structures and/or perforate visceral peritoneum

**N - Regional lymph nodes**
Nx - Regional lymph nodes cannot be assessed
N0 - No regional lymph node metastasis
N1 - Metastasis in 1 to 3 regional lymph nodes
N2 - Metastasis in 4 or more regional lymph nodes

**M - Distant metastasis**
Mx - Distant metastasis cannot be assessed
M0 - No distant metastasis
M1 - Distant metastasis
