# Supplementary material for: Expression of Myoglobin in Normal and Cancer Brain Tissues: Correlation With Hypoxia Markers
Source: Front Oncol. 2021 Apr 30;11:590771. doi: 10.3389/fonc.2021.590771 (PMC8120281; doi:10.3389/fonc.2021.590771)
Supplement: Supplementary Document 2 — Brain tumor and normal tissue microarray. [file DataSheet_2.pdf]

GL804 : Brain tumor and normal tissue array with grade and TNM data, 40 cases/80 cores

Microarray Panel	Brain tumor and normal tissue microarray, containing 26 cases of astrocytoma, 4 oligodendroglioma, 5 each of adjacent normal tissue and normal tissue, duplicate cores per case, with pathology grade and clinical TNM information
Cores	80
Cases	40
Row number	8
Column number	10
Core Diameter (mm)	1.5
Thickness (µm)	5
QA/QC	Anti-Cytokeratin (CK) confirmed
Tissue Array Type	FFPE
Species	Human
Applications	Routine histology procedures including Immunohistochemistry (IHC) and In Situ Hybridization (ISH), protocols which can be found at our support page.
Notes	1. TMA slides were sectioned and stored at 4°C and may not be fresh cut, but still suitable for IHC. Please request fresh cut if experiment involves phospho-specific antibodies, RNA studies, FISH or ISH, etc. A minimum of 3 slides per TMA must be purchased to cover the cost of trimming for fresh sectioning. 2. Most TMA slides were not coated with an extra layer of paraffin (tissue cores can be easily seen on the glass). **To prevent tissue detachment during antigen retrieval, unbaked slides must be baked for at least 30 to 120 minutes at 60°C**. before putting into xylene for de-paraffinization. Baked slides were sent out baked for 2 hours.<br>In the following specsheet,"*" means invalid core; "-" means no applicable or negative in IHC markers.

GL804 H&E

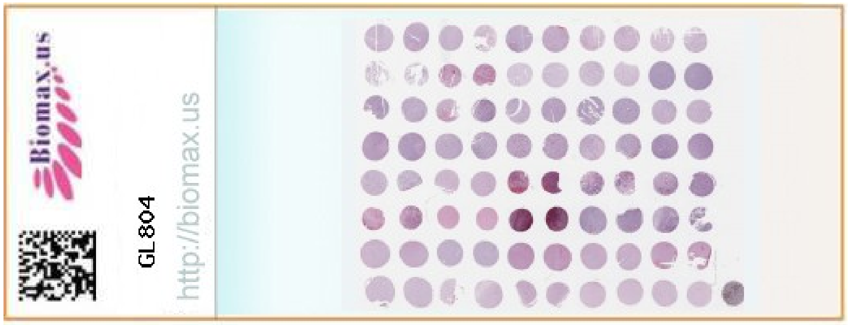

Mouseover and click individual cores to view high resolution images.

US Biomax, Inc. GL804 (serial)

	1	2	3	4	5	6	7	8	9	10	
A	Bra	Bra	Bra	Bra	Bra	Bra	Bra	Bra	Bra	Bra	
B	Bra	Bra	Bra	Bra	Bra	Bra	Bra	Bra	Bra	Bra	
C	Bra	Bra	Bra	Bra	Bra	Bra	Bra	Bra	Bra	Bra	
D	Bra	Bra	Bra	Bra	Bra	Bra	Bra	Bra	Bra	Bra	
E	Bra	Bra	Bra	Bra	Bra	Bra	Bra	Bra	Bra	Bra	
F	Bra	Bra	Bra	Bra	Bra	Bra	Bra	Bra	Bra	Bra	
G	Bra	Bra	Bra	Bra	Bra	Bra	Bra	Bra	Bra	Bra	
H	Bra	Bra	Bra	Bra	Bra	Bra	Bra	Bra	Bra	Bra	Ski

Legend: **Bra** - Brain
● - Malignant tumor, ● - NAT, ● - Normal tissue

, tissue IDs are available in exported Excel files.

Pos.	No.	Age	Sex	Organ/Anatomic Site	Pathology diagnosis	TNM	Grade	Stage	Type	Image
A1	1	44	F	Brain	Astrocytoma (brain tissue)	T1N0M0	-		malignant	
A2	2	44	F	Brain	Astrocytoma	T1N0M0	1--2		malignant	
A3	3	30	M	Brain	Astrocytoma	T1N0M0	1		malignant	
A4	4	30	M	Brain	Astrocytoma	T1N0M0	1		malignant	
A5	5	51	M	Brain	Astrocytoma	T1N0M0	2		malignant	
A6	6	51	M	Brain	Astrocytoma	T1N0M0	2		malignant	
A7	7	16	F	Brain	Astrocytoma	T1N0M0	2		malignant	
A8	8	16	F	Brain	Astrocytoma	T1N0M0	2		malignant	
A9	9	15	F	Brain	Astrocytoma	T1N0M0	2		malignant	
A10	10	15	F	Brain	Astrocytoma	T1N0M0	2		malignant	
B1	11	37	M	Brain	Astrocytoma	-	2		malignant	
B2	12	37	M	Brain	Astrocytoma	-	2		malignant	

B3	13	10	M	Brain	Astrocytoma	T1N0M0	2		malignant	
B4	14	10	M	Brain	Astrocytoma	T1N0M0	2		malignant	
B5	15	33	M	Brain	Astrocytoma	T1N0M0	1		malignant	
B6	16	33	M	Brain	Astrocytoma	T1N0M0	2		malignant	
B7	17	62	M	Brain	Astrocytoma	T1N0M0	2		malignant	
B8	18	62	M	Brain	Astrocytoma (brain tissue)	T1N0M0	-		malignant	
B9	19	40	F	Brain	Astrocytoma	T2N0M0	2		malignant	
B10	20	40	F	Brain	Astrocytoma	T2N0M0	2		malignant	
C1	21	12	M	Brain	Protoplasmic astrocytoma	T1N0M0	2		malignant	
C2	22	12	M	Brain	Protoplasmic astrocytoma	T1N0M0	2		malignant	
C3	23	35	M	Brain	Astrocytoma	T1M0	3		malignant	
C4	24	35	M	Brain	Astrocytoma	T1M0	3		malignant	
C5	25	57	M	Brain	Astrocytoma	T2N0M0	2		malignant	
C6	26	57	M	Brain	Astrocytoma	T2N0M0	2		malignant	
C7	27	39	M	Brain	Astrocytoma	T3N0M0	2		malignant	
C8	28	39	M	Brain	Astrocytoma	T3N0M0	2		malignant	
C9	29	30	F	Brain	Astrocytoma	T2N0M0	2		malignant	
C10	30	30	F	Brain	Astrocytoma	T2N0M0	2		malignant	
D1	31	60	F	Brain	Astrocytoma	T1N0M0	2--3		malignant	
D2	32	60	F	Brain	Astrocytoma	T1N0M0	2--3		malignant	
D3	33	31	M	Brain	Astrocytoma	T2N0M0	2		malignant	
D4	34	31	M	Brain	Astrocytoma	T2N0M0	2		malignant	
D5	35	53	F	Brain	Atypical astrocytoma	T2N0M0	3		malignant	
D6	36	53	F	Brain	Atypical astrocytoma	T2N0M0	3		malignant	
D7	37	40	F	Brain	Glioblastoma	T2N0M0	3--4		malignant	
D8	38	40	F	Brain	Glioblastoma	T2N0M0	3--4		malignant	
D9	39	47	F	Brain	Glioblastoma	T2N0M0	3--4		malignant	
D10	40	47	F	Brain	Glioblastoma	T2N0M0	3--4		malignant	
E1	41	16	F	Brain	Glioblastoma	T2N0M0	4		malignant	
E2	42	16	F	Brain	Glioblastoma	T2N0M0	4		malignant	
E3	43	12	M	Brain	Glioblastoma (brain tissue)	T1N0M0	-		malignant	
E4	44	12	M	Brain	Glioblastoma (brain tissue)	T1N0M0	-		malignant	
E5	45	49	F	Brain	Glioblastoma	-	4		malignant	
E6	46	49	F	Brain	Glioblastoma	-	4		malignant	
E7	47	39	M	Brain	Glioblastoma	T2N0M0	4		malignant	
E8	48	39	M	Brain	Glioblastoma	T2N0M0	4		malignant	
E9	49	31	F	Brain	Glioblastoma	-	4		malignant	
E10	50	31	F	Brain	Glioblastoma	-	4		malignant	
F1	51	39	M	Brain	Glioblastoma	T1N0M0	4		malignant	
F2	52	39	M	Brain	Glioblastoma	T1N0M0	4		malignant	
F3	53	39	M	Brain	Oligodendroglioma (sparse)	T1N0M0	1		malignant	
F4	54	39	M	Brain	Oligodendroglioma (brain tissue)	T1N0M0	-		malignant	
F5	55	56	F	Brain	Oligodendroglioma	T1N0M0	2		malignant	
F6	56	56	F	Brain	Oligodendroglioma	T1N0M0	2		malignant	
F7	57	52	M	Brain	Oligodendroglioma	T2N0M0	2--3		malignant	
F8	58	52	M	Brain	Oligodendroglioma	T2N0M0	2--3		malignant	
F9	59	17	M	Brain	Anaplastic oligodendroglioma	T1N0M0	-		malignant	
F10	60	17	M	Brain	Anaplastic oligodendroglioma	T1N0M0	-		malignant	
G1	61	52	M	Brain	Cancer adjacent normal brain tissue	-	-		NAT	
G2	62	52	M	Brain	Cancer adjacent normal brain tissue	-	-		NAT	
G3	63	39	M	Brain	Cancer adjacent normal brain tissue	-	-		NAT	
G4	64	39	M	Brain	Cancer adjacent normal brain tissue	-	-		NAT	
G5	65	37	F	Brain	Cancer adjacent normal brain tissue	-	-		NAT	
G6	66	37	F	Brain	Cancer adjacent normal brain tissue	-	-		NAT	
G7	67	68	M	Brain	Cancer adjacent normal brain tissue	-	-		NAT	
G8	68	68	M	Brain	Cancer adjacent normal brain tissue	-	-		NAT	
G9	69	29	F	Brain	Cancer adjacent normal brain tissue	-	-		NAT	
G10	70	29	F	Brain	Cancer adjacent normal brain tissue	-	-		NAT	
H1	71	21	F	Brain	Normal brain tissue	-	-		normal	
H2	72	21	F	Brain	Normal brain tissue	-	-		normal	

H3	73	21	F	Brain	Normal brain tissue	-	-		normal	
H4	74	21	F	Brain	Normal brain tissue	-	-		normal	
H5	75	40	F	Brain	Normal brain tissue	-	-		normal	
H6	76	40	F	Brain	Normal brain tissue	-	-		normal	
H7	77	40	M	Brain	Normal brain tissue	-	-		normal	
H8	78	40	M	Brain	Normal brain tissue	-	-		normal	
H9	79	40	M	Brain	Normal brain tissue	-	-		normal	
H10	80	40	M	Brain	Normal brain tissue	-	-		normal	
-	0	58	M	Skin	Malignant melanoma (tissue marker)		-		Malignant	

TNM grading:

T - Primary tumor
Tx - Primary tumor cannot be assessed
T0 - No evidence of primary tumor
Tis - Carcinoma in situ; intraepithelial or invasion of lamina propria
T1 - Tumor invades submucosa
T2 - Tumor invades muscularis propria
T3 - Tumor invades through muscularis propria into subserosa or into non-peritonealized pericolic or perirectal tissues.
T4 - Tumor directly invades other organs or structures and/or perforate visceral peritoneum

N - Regional lymph nodes
Nx - Regional lymph nodes cannot be assessed
N0 - No regional lymph node metastasis
N1 - Metastasis in 1 to 3 regional lymph nodes
N2 - Metastasis in 4 or more regional lymph nodes

M - Distant metastasis
Mx - Distant metastasis cannot be assessed
M0 - No distant metastasis
M1 - Distant metastasis
