# Supplementary material for: Expression of Myoglobin in Normal and Cancer Brain Tissues: Correlation With Hypoxia Markers
Source: Front Oncol. 2021 Apr 30;11:590771. doi: 10.3389/fonc.2021.590771 (PMC8120281; doi:10.3389/fonc.2021.590771)
Supplement: Supplementary Document 3 — Glioblastoma multiforme and normal brain tissue microarray. [file DataSheet_3.pdf]

GL805a : Glioblastoma multiforme (GBM) tissue array with normal brain tissue as control, including pathology grade, 40 cases/ 80 cores, replace

Microarray Panel	Brain tumor tissue microarray with normal tissue as control, containing 35 cases of glioblastoma, 2 adjacent brain tissue and 3 normal brain tissue, duplicated cores per case
Cores	80
Cases	40
Row number	8
Column number	10
Core Diameter (mm)	1.5
Thickness (µm)	5
Tissue Array Type	FFPE
Species	Human
Applications	Routine histology procedures including Immunohistochemistry (IHC) and In Situ Hybridization (ISH), protocols which can be found at our support page.
Notes	1. TMA slides were sectioned and stored at 4°C and may not be fresh cut, but still suitable for IHC. Please request fresh cut if experiment involves phospho-specific antibodies, RNA studies, FISH or ISH, etc. A minimum of 3 slides per TMA must be purchased to cover the cost of trimming for fresh sectioning. 2. Most TMA slides were not coated with an extra layer of paraffin (tissue cores can be easily seen on the glass). **To prevent tissue detachment during antigen retrieval, unbaked slides must be baked for at least 30 to 120 minutes at 60°C**. before putting into xylene for de-paraffinization. Baked slides were sent out baked for 2 hours.<br>In the following specsheet,"*" means invalid core; "-" means no applicable or negative in IHC markers.

GL805a H&E

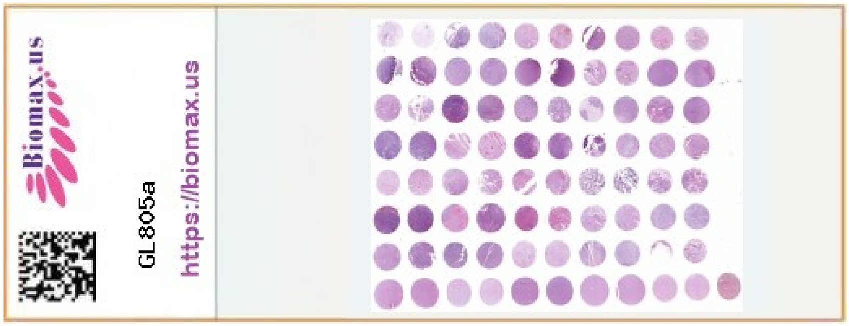

Mouseover and click individual cores to view high resolution images.

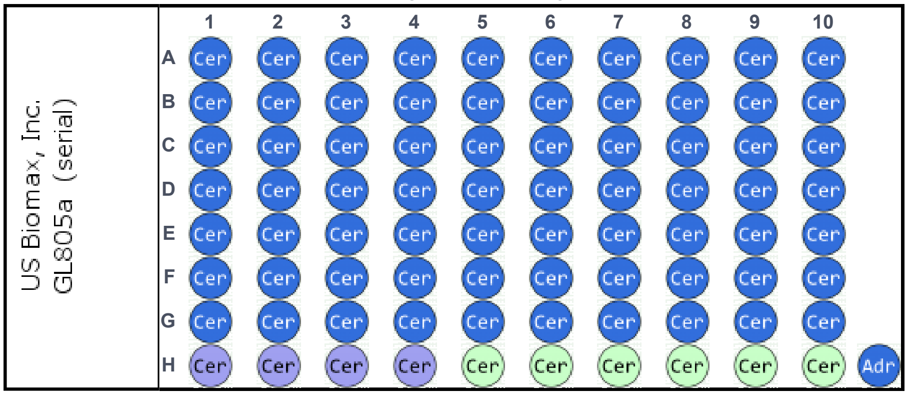

Legend: **Cer** - Cerebrum
- Malignant tumor, - NAT, - Normal tissue

, tissue IDs are available in exported Excel files.

Pos.	No.	Age	Sex	Organ/Anatomic Site	Pathology diagnosis	TNM	Grade	Stage	Type	Image
A1	1	64	M	Cerebrum	Glioblastoma (necrosis tissue)		-		malignant	
A2	2	64	M	Cerebrum	Glioblastoma		3--4		malignant	
A3	3	21	M	Cerebrum	Glioblastoma		4		malignant	
A4	4	21	M	Cerebrum	Glioblastoma		4		malignant	
A5	5	33	F	Cerebrum	Glioblastoma		4		malignant	
A6	6	33	F	Cerebrum	Glioblastoma		4		malignant	
A7	7	59	M	Cerebrum	Glioblastoma		4		malignant	
A8	8	59	M	Cerebrum	Glioblastoma		4		malignant	
A9	9	17	M	Cerebrum	Glioblastoma		3--4		malignant	
A10	10	17	M	Cerebrum	Glioblastoma		3--4		malignant	
B1	11	53	M	Cerebrum	Glioblastoma		4		malignant	
B2	12	53	M	Cerebrum	Glioblastoma		4		malignant	
B3	13	11	F	Cerebrum	Glioblastoma		4		malignant	
B4	14	11	F	Cerebrum	Glioblastoma		4		malignant	

B5	15	47	M	Cerebrum	Glioblastoma		4		malignant
B6	16	47	M	Cerebrum	Glioblastoma		4		malignant
B7	17	38	M	Cerebrum	Glioblastoma		4		malignant
B8	18	38	M	Cerebrum	Glioblastoma (sparse)		4		malignant
B9	19	43	M	Cerebrum	Glioblastoma		4		malignant
B10	20	43	M	Cerebrum	Glioblastoma		4		malignant
C1	21	19	M	Cerebrum	Glioblastoma		4		malignant
C2	22	19	M	Cerebrum	Glioblastoma		4		malignant
C3	23	31	F	Cerebrum	Glioblastoma		4		malignant
C4	24	31	F	Cerebrum	Glioblastoma		4		malignant
C5	25	20	M	Cerebrum	Glioblastoma		4		malignant
C6	26	20	M	Cerebrum	Glioblastoma		4		malignant
C7	27	6	F	Cerebrum	Glioblastoma		4		malignant
C8	28	6	F	Cerebrum	Glioblastoma		4		malignant
C9	29	63	M	Cerebrum	Glioblastoma		4		malignant
C10	30	63	M	Cerebrum	Glioblastoma		4		malignant
D1	31	23	M	Cerebrum	Glioblastoma		4		malignant
D2	32	23	M	Cerebrum	Glioblastoma		4		malignant
D3	33	59	M	Cerebrum	Glioblastoma		4		malignant
D4	34	59	M	Cerebrum	Glioblastoma		4		malignant
D5	35	47	M	Cerebrum	Glioblastoma		4		malignant
D6	36	47	M	Cerebrum	Glioblastoma		4		malignant
D7	37	26	F	Cerebrum	Glioblastoma		4		malignant
D8	38	26	F	Cerebrum	Glioblastoma		4		malignant
D9	39	26	M	Cerebrum	Glioblastoma		4		malignant
D10	40	26	M	Cerebrum	Glioblastoma		4		malignant
E1	41	42	M	Cerebrum	Glioblastoma		4		malignant
E2	42	42	M	Cerebrum	Glioblastoma		4		malignant
E3	43	44	M	Cerebrum	Glioblastoma		4		malignant
E4	44	44	M	Cerebrum	Glioblastoma		4		malignant
E5	45	32	F	Cerebrum	Glioblastoma		4		malignant
E6	46	32	F	Cerebrum	Glioblastoma		4		malignant
E7	47	52	M	Cerebrum	Glioblastoma		4		malignant
E8	48	52	M	Cerebrum	Glioblastoma		4		malignant
E9	49	22	F	Cerebrum	Glioblastoma (astrocytoma)		2		malignant
E10	50	22	F	Cerebrum	Glioblastoma (astrocytoma)		2		malignant
F1	51	34	M	Cerebrum	Glioblastoma		4		malignant
F2	52	34	M	Cerebrum	Glioblastoma		4		malignant
F3	53	66	F	Cerebrum	Glioblastoma (astrocytoma)		2		malignant
F4	54	66	F	Cerebrum	Glioblastoma (astrocytoma)		2		malignant
F5	55	50	F	Cerebrum	Glioblastoma		4		malignant
F6	56	50	F	Cerebrum	Glioblastoma		4		malignant
F7	57	41	F	Cerebrum	Glioblastoma		4		malignant
F8	58	41	F	Cerebrum	Glioblastoma		4		malignant
F9	59	58	F	Cerebrum	Glioblastoma		4		malignant
F10	60	58	F	Cerebrum	Glioblastoma		4		malignant
G1	61	39	M	Cerebrum	Glioblastoma		4		malignant
G2	62	39	M	Cerebrum	Glioblastoma		4		malignant
G3	63	70	F	Cerebrum	Glioblastoma		4		malignant
G4	64	70	F	Cerebrum	Glioblastoma		4		malignant
G5	65	27	F	Cerebrum	Glioblastoma		4		malignant
G6	66	27	F	Cerebrum	Glioblastoma		4		malignant
G7	67	49	M	Cerebrum	Glioblastoma		4		malignant
G8	68	49	M	Cerebrum	Glioblastoma		4		malignant
G9	69	75	M	Cerebrum	Glioblastoma		4		malignant
G10	70	75	M	Cerebrum	Glioblastoma		4		malignant
H1	71	52	M	Cerebrum	Cerebral tissue		-		NAT
H2	72	52	M	Cerebrum	Cerebral tissue		-		NAT
H3	73	30	M	Cerebrum	Cerebral tissue		-		NAT
H4	74	30	M	Cerebrum	Cerebral tissue		-		NAT

H5	75	38	F	Cerebrum	Cerebral tissue		-		normal	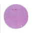
H6	76	38	F	Cerebrum	Cerebral tissue		-		normal	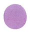
H7	77	50	F	Cerebrum	Cerebral tissue		-		normal	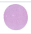
H8	78	50	F	Cerebrum	Cerebral tissue		-		normal	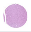
H9	79	16	M	Cerebrum	Cerebral tissue		-		normal	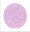
H10	80	16	M	Cerebrum	Cerebral tissue		-		normal	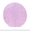
-	0	42	M	Adrenal gland	Pheochromocytoma (tissue marker)		-		Malignant	

TNM grading:

T - Primary tumor

Tx - Primary tumor cannot be assessed
T0 - No evidence of primary tumor
Tis - Carcinoma in situ; intraepithelial or invasion of lamina propria
T1 - Tumor invades submucosa
T2 - Tumor invades muscularis propria
T3 - Tumor invades through muscularis propria into subserosa or into non-peritonealized pericolic or perirectal tissues.
T4 - Tumor directly invades other organs or structures and/or perforate visceral peritoneum

N - Regional lymph nodes

Nx - Regional lymph nodes cannot be assessed
N0 - No regional lymph node metastasis
N1 - Metastasis in 1 to 3 regional lymph nodes
N2 - Metastasis in 4 or more regional lymph nodes

M - Distant metastasis

Mx - Distant metastasis cannot be assessed
M0 - No distant metastasis
M1 - Distant metastasis
